# Supplementary material for: Optimizing the detection of emerging infections using mobility-based spatial sampling
Source: Int J Appl Earth Obs Geoinf. 2024 Jul;131:103949. doi: 10.1016/j.jag.2024.103949 (PMC11234252; doi:10.1016/j.jag.2024.103949)
Supplement: Supplementary Data 1 [file mmc1.docx]

# Supplementary information for “Optimizing the detection of emerging infections using mobility-based spatial sampling”

Die Zhang^a,b,#^, Yong Ge^b,^^c,d*,#^, Jianghao Wang^b,d,#^, Haiyan Liu^e,#^, Wen-Bin Zhang^b,f^, Xilin Wu^b,d^, Gerard B. M. Heuvelink^g,h^, Chaoyang Wu^d,i^, Juan Yang^j,k^, Nick W. Ruktanonchai^f,l^, Sarchil H. Qader^f,m^, Corrine W. Ruktanonchai^f,l^, Eimear Cleary^f^, Yongcheng Yao^f,n^, Jian Liu^e^, Chibuzor C. Nnanatu^f^, Amy Wesolowski^o^, Derek A.T. Cummings^p^, Andrew J. Tatem^f,*^, Shengjie Lai^f,k,q,*^

**Affiliations:**

^a^School of Geography and Environment, Jiangxi Normal University, Nanchang, China;

^b^State Key Laboratory of Resources and Environmental Information System, Institute of Geographic Sciences & Natural Resources Research, Chinese Academy of Sciences, Beijing, China;

^c^Key Laboratory of Poyang Lake Wetland and Watershed Research Ministry of Education, Jiangxi Normal University, Nanchang, China;

^d^University of Chinese Academy of Sciences, Beijing, China;

^e^Ocean Data Center, Southern Marine Science and Engineering Guangdong Laboratory (Zhuhai), Zhuhai, China;

^f^WorldPop, School of Geography and Environmental Science, University of Southampton, Southampton, UK;

^g^ISRIC - World Soil Information, Wageningen, the Netherlands;

^h^Soil Geography and Landscape Group, Wageningen University, Wageningen, the Netherlands;

^i^The Key Laboratory of Land Surface Pattern and Simulation, Institute of Geographical Sciences and Natural Resources Research, Chinese Academy of Sciences, Beijing, China;

^j^School of Public Health, Fudan University, Key Laboratory of Public Health Safety, Ministry of Education, Shanghai, China

^k^Shanghai Institute of Infectious Disease and Biosecurity, Fudan University, Shanghai, China

^l^Population Health Sciences, Virginia Tech, Blacksburg, VA, USA;

^m^Natural Resources Department, College of Agricultural Engineering Sciences, University of Sulaimani; Sulaimani 334, Kurdistan Region, Iraq;

^n^School of Mathematics and Statistics, Zhengzhou Normal University, Zhengzhou, China;

^o^Department of Epidemiology, Johns Hopkins Bloomberg School of Public Health, Baltimore, MD, USA;

^p^Department of Biology and Emerging Pathogens Institute, University of Florida, Gainesville, FL, USA;

^q^Institute for Life Sciences, University of Southampton, Southampton, UK.

^#^Authors contributed equally to this work.

**^*^Correspondence:**

Shengjie Lai, Email: [Shengjie.Lai@soton.ac.uk](mailto:Shengjie.Lai@soton.ac.uk)

Yong Ge, Email: [gey@lreis.ac.cn](mailto:gey@lreis.ac.cn)

Andrew J. Tatem, Email: [A.J.Tatem@soton.ac.uk](mailto:A.J.Tatem@soton.ac.uk)

# Supplementary Figures and Tables


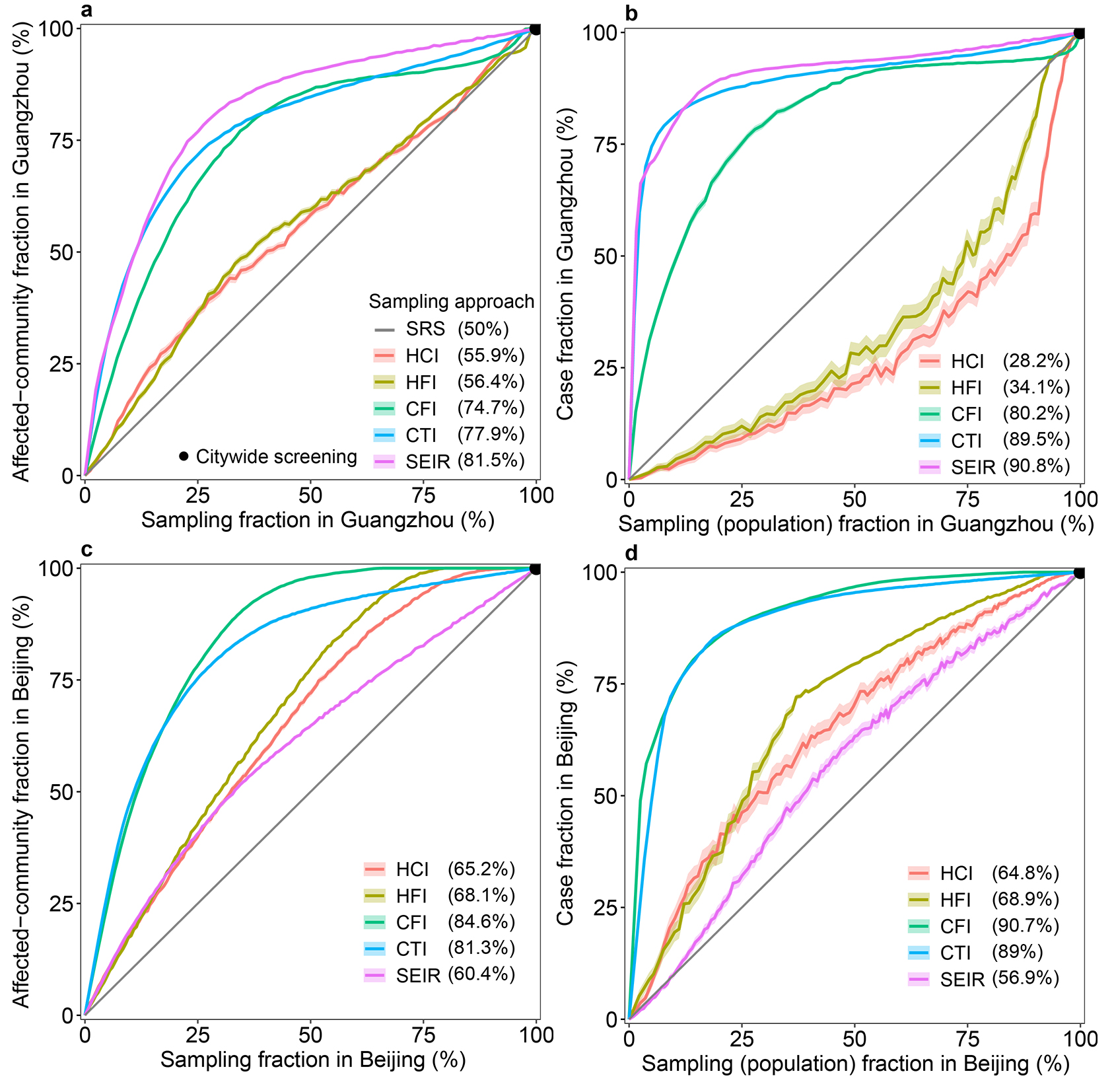


**Fig. S1. Performance of mobility-based spatial sampling approaches in detecting COVID-19 affected communities and cases at varying sample sizes.** Four mobility-based spatial sampling approaches (HCI - human contact intensity; HFI - human flow intensity; CFI - case flow intensity; CTI - case transmission intensity) and an epidemiological model (SEIR) were evaluated. Communities with high infection risk ($\rho_{i}$) were sampled by ranking community-level $\rho_{i}$ from high to low, excluding the simple random sampling (SRS) method. The x-axis in **a** and **c** represents the proportion of sampled communities over the total number of communities in Guangzhou and Beijing, respectively. In **b** and **d**, the x-axis denotes the fraction of sampled populations among the total populations. The y-axis in **a** and **c** represents the proportion of affected communities sampled over the total communities with COVID-19 cases in Guangzhou and Beijing. In **b** and **d**, the y-axis displays the proportion of cases detected by different sampling approaches among the total cases. The percentage in the legend indicates the area under each curve, reflecting the average performance of each sampling approach with different sample sizes. The black dot at the upper right corner of each panel represents citywide screening for the entire population, assuming the test can detect all infected people in the city. Shaded regions denote 95% confidence intervals.


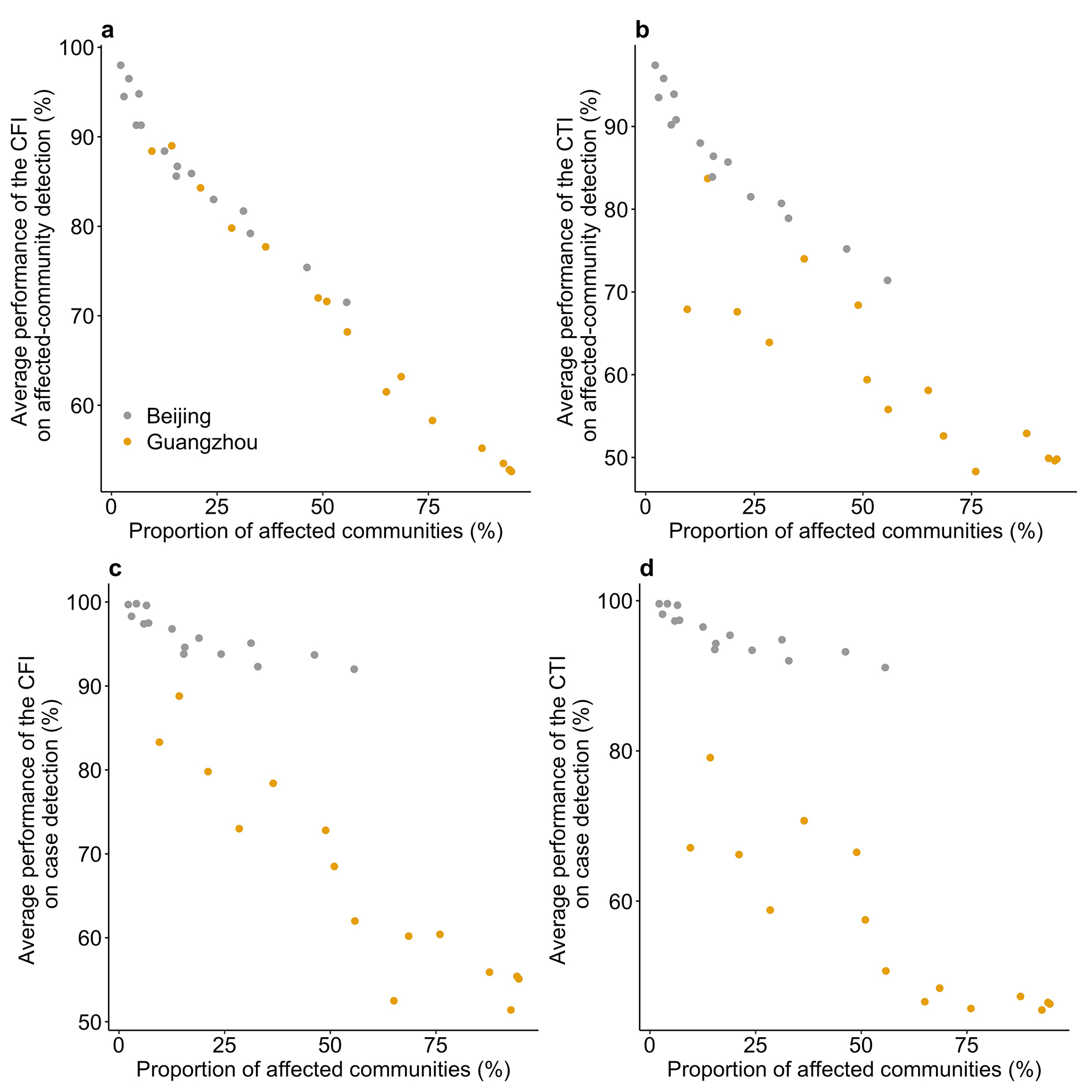


**Fig. S2**. **Average performance of case flow intensity (CFI) and case transmission intensity (CTI) sampling in identifying affected communities/cases in Guangzhou and Beijing under various outbreak and data scenarios.** The x-axis represents the ratio of affected communities under simulated scenarios over the total communities in a city. The average accuracy of CFI and CTI for identifying affected communities/cases was reduced by increasing the geographic extent of epidemic transmissions across communities within a city.


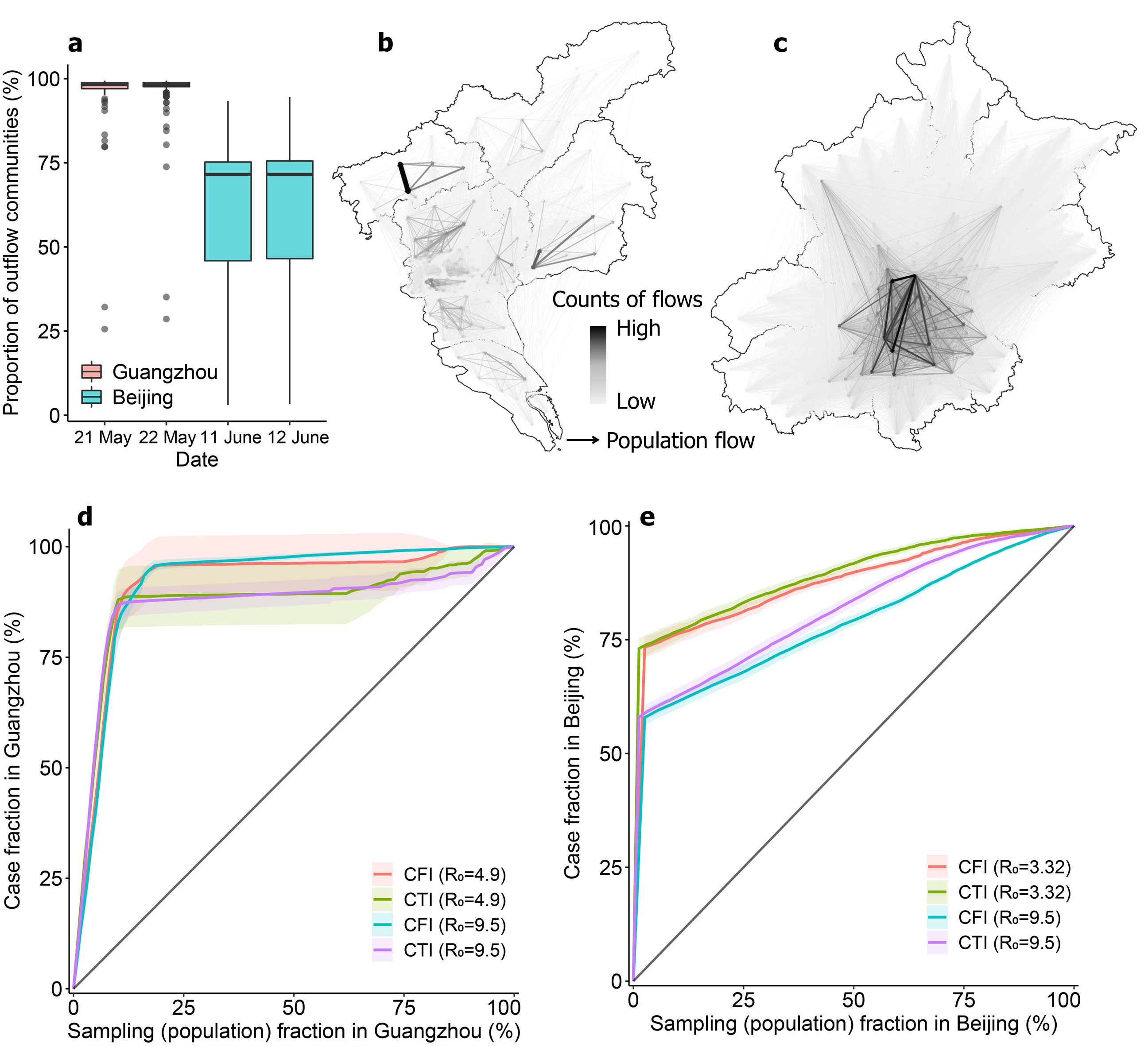


**Fig. S3. Performance of mobility-based spatial sampling in simulated outbreaks under the exchange of mobility patterns between Guangzhou and Beijing.** Outbreaks were generated using a travel network-based epidemiological model based on simulated inter-community population flow data. **a,** Proportion of the cumulative number of distinct communities that individuals from a particular community visited within a single day, computed based on actual population flow data in the two cities. **b,** Human mobility pattern in Guangzhou created in terms of Beijing’s real-world mobility characteristics. **c,** Simulated mobility pattern in Beijing according to Guangzhou’s actual mobility characteristics. Directed lines represent inter-community origin-destination travel networks on 21-22 May 2021 in Guangzhou and 11-12 June 2020 in Beijing, respectively. The color and width of an edge depict the volume of an inter-community flow. Based on simulated epidemics under two basic reproduction numbers (R_0_) types, two optimized mobility-based spatial sampling approaches (CFI - case flow intensity and CTI - case transmission intensity) were assessed. The x-axis in **d** and **e** presents the fraction of sampled populations among the total populations in a city, and the y-axis shows the proportion of cases detected by different sampling approaches. The diagonal line in each panel denotes the performance of simple random sampling. Shaded regions represent the 95% confidence intervals.


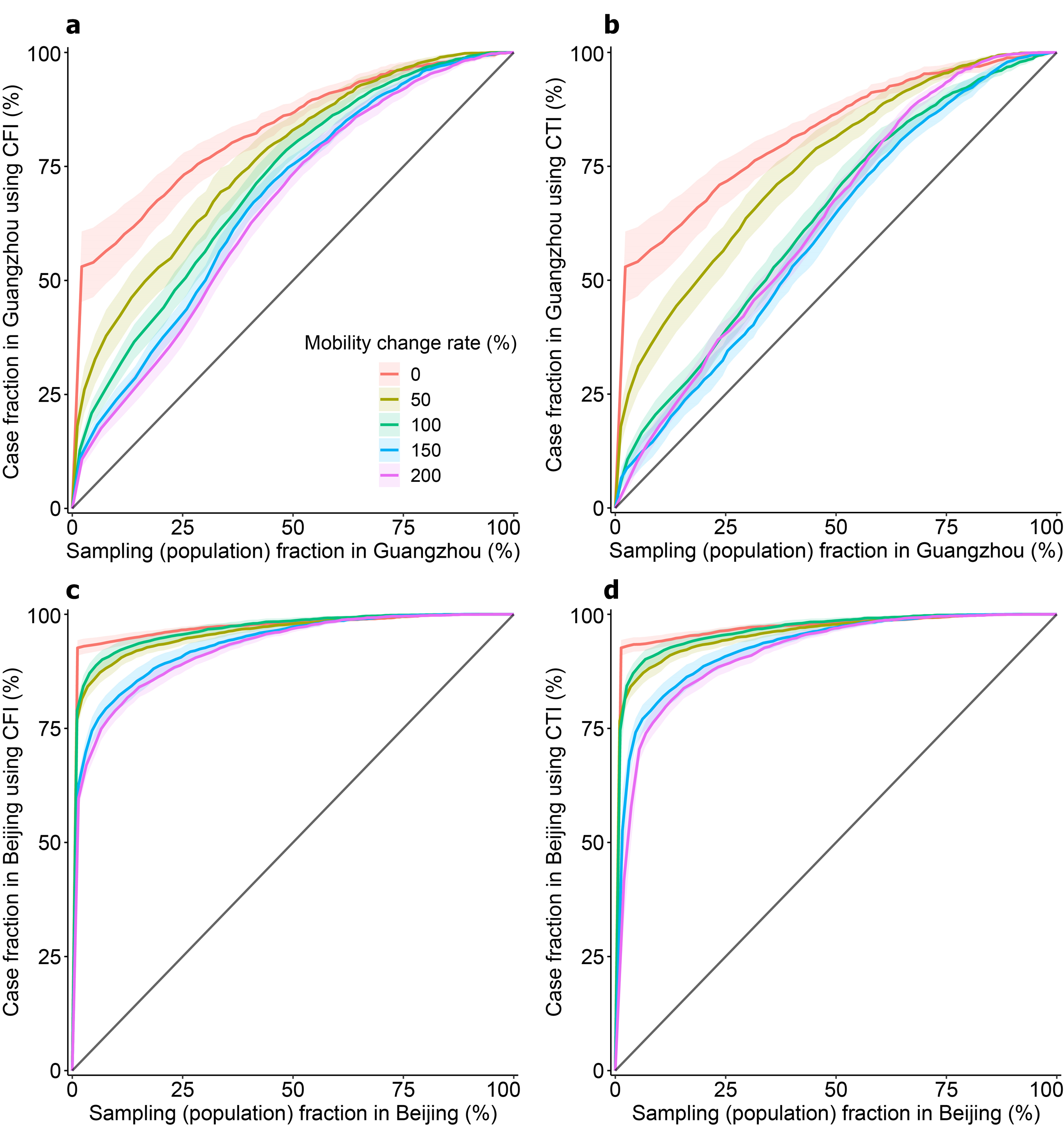


**Fig. S4. Performance of mobility-based spatial sampling in simulated outbreaks under various levels of population mobility in Guangzhou and Beijing.** Outbreaks were generated using a travel network-based epidemiological model based on simulated inter-community flow data. The levels of population mobility changed for the top 20% of real-world inter-community flows within a city, and the change rates were 0%, 50%, 150%, and 200%, respectively. Based on simulated epidemics with R_0_ equal to 4.9 in Guangzhou and 3.32 in Beijing, two optimized mobility-based spatial sampling approaches (CFI - case flow intensity and CTI - case transmission intensity) were assessed. The x-axis represents the fraction of sampled populations among the total population in a city, and the y-axis shows the proportion of cases detected by different sampling approaches. The diagonal line in each panel denotes the performance of simple random sampling, and shaded regions represent the 95% confidence intervals.


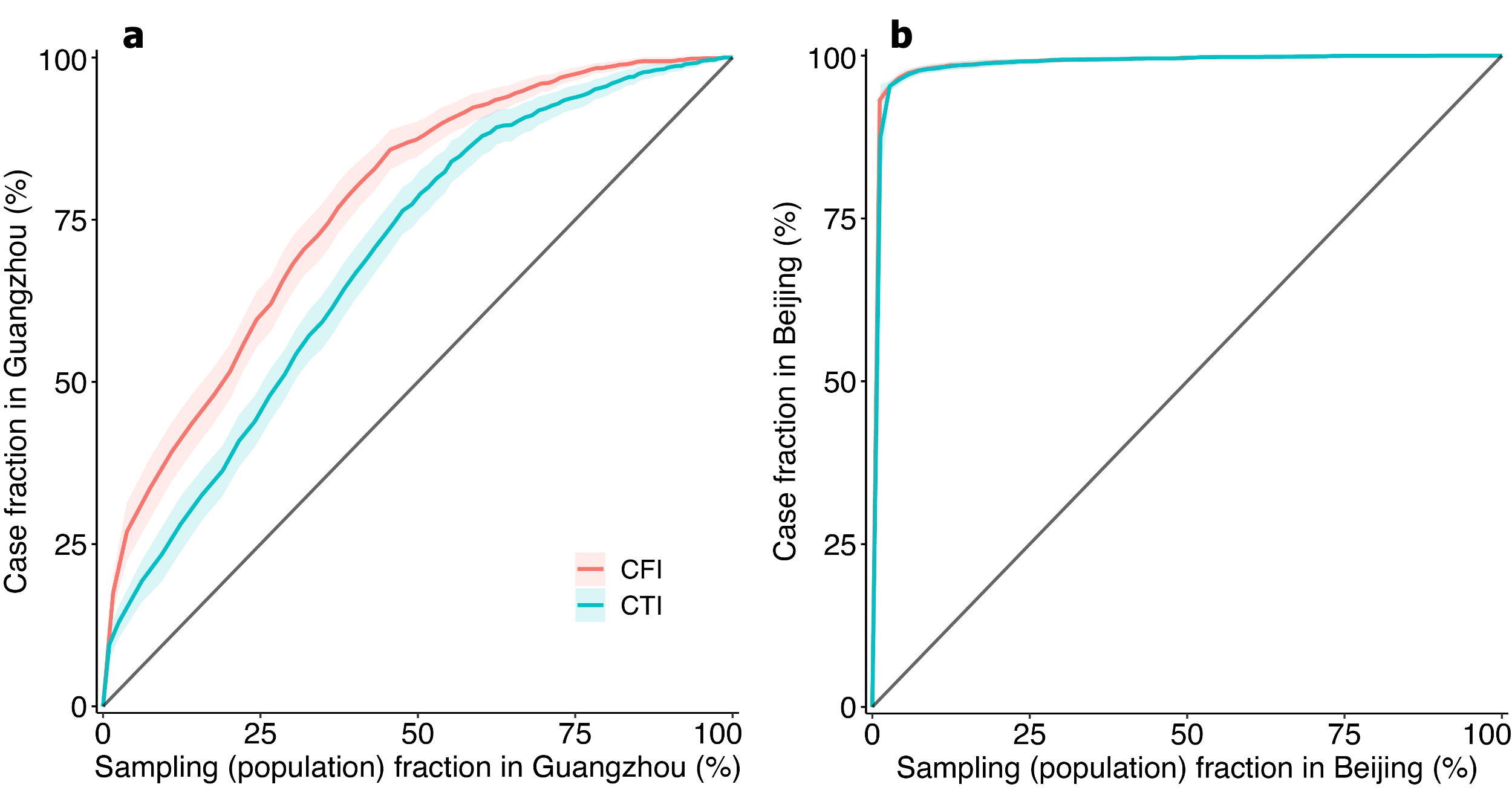


**Fig. S5. Performance of mobility-based spatial sampling in simulated outbreaks under various epidemiological parameter combinations.** Outbreaks were simulated using a travel network-based epidemiological model based on daily parameters. The parameters were randomly selected per day from a given range: basic reproduction numbers (R_0_) changing daily from 1.4 to 6.5, the incubation period was between 4.1 and 7, and the infectious period generated by mean and variance. Based on simulated epidemics with random parameter combinations, two optimized mobility-based spatial sampling approaches (CFI - case flow intensity and CTI - case transmission intensity) were assessed. The x-axis in **a** and **b** represents the fraction of sampled populations among the total population in Guangzhou and Beijing, respectively, and the y-axis shows the proportion of cases detected by different sampling approaches. The diagonal line in each panel denotes the performance of the simple random sampling, and shaded regions represent the 95% confidence intervals.


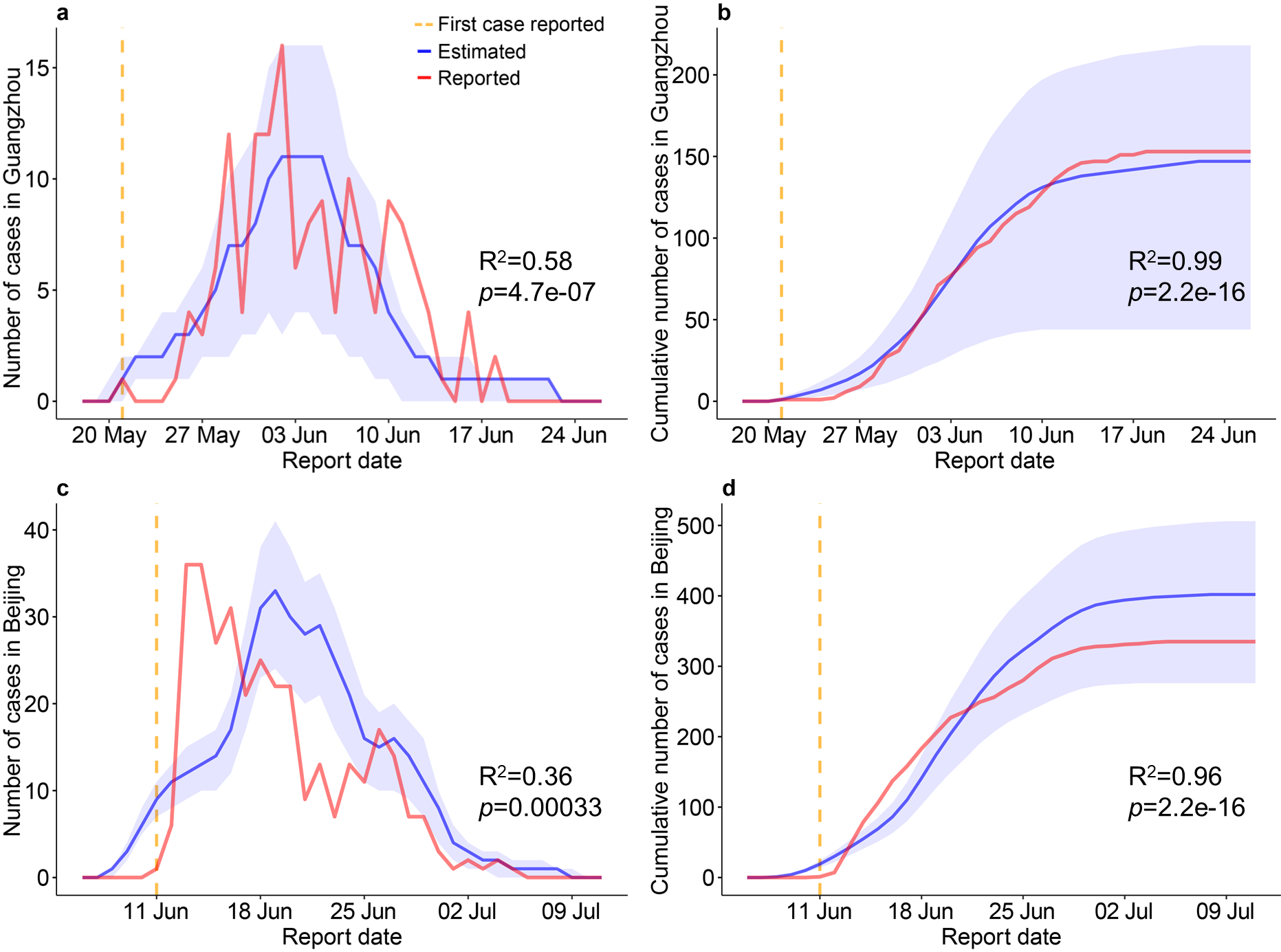


**Fig. S6**. **Estimated epidemic curves using the constructed SEIR model and reported curves for the read-world COVID-19 outbreak in Guangzhou and Beijing.** **a** and **c** are the estimated number of daily new cases in the two cities, and **b** and **d** are the cumulative numbers of daily cases. Estimated transmissions are presented as the mean (solid blue lines) and IQR (shading) of 500 simulations. Red lines denote reported transmissions during the outbreak in Guangzhou and Beijing, and yellow dotted lines are the day the first case was reported. The report date was determined according to the infection date and time lags from being infected to reported.


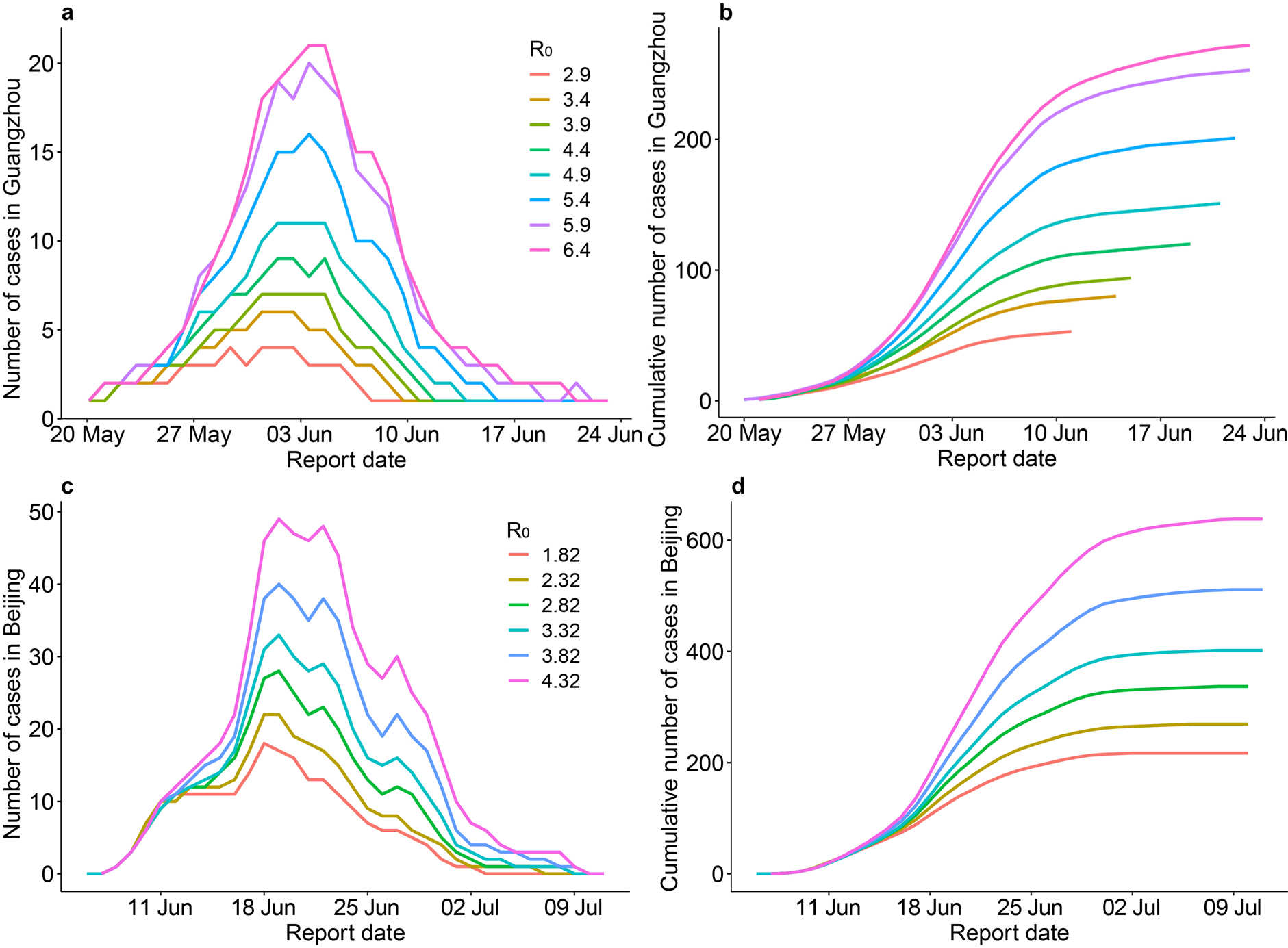


**Fig. S7.** **Sensitivity of estimates of the real-world COVID-19 outbreak in Guangzhou and Beijing for various values of R_0_.** All other parameters and input data were the same as the baseline model with R_0_=4.9 in Guangzhou and R_0_=3.32 in Beijing. Colored lines represent estimated curves for various values of R_0_. Estimated epidemic curves from the constructed SEIR were sensitive to the R_0_, where the peaks of epidemics were higher and later under a higher R_0_ value.

**Table S1. Number of cases in each affected community during COVID-19 outbreaks in Guangzhou and Beijing.** There were 152 cases in 16 communities of Guangzhou and 368 cases in 52 communities of Beijing, respectively.

| **Community name** | **Number of cases** | **Community name** | **Number of cases** |
| --- | --- | --- | --- |
| **Guangzhou (21 May**–**18 June 2021)** | | | |
| Baihedong Subdistrict | 91 | Longjin Subdistrict | 2 |
| Zhongnan Subdistrict | 29 | Taihe Town | 1 |
| Zhujiang Subdistrict | 10 | Changgang Subdistrict | 1 |
| Ruibao Subdistrict | 4 | Haichuang Subdistrict | 1 |
| Dongjiao Subdistrict | 3 | [Nanhuaxi Subdistrict](https://en.wikipedia.org/w/index.php?title=Nanhuaxi_Subdistrict&action=edit&redlink=1) | 1 |
| Dashi Subdistrict | 2 | [Beijing Subdistrict](https://en.wikipedia.org/wiki/Beijing_Subdistrict,_Guangzhou) | 1 |
| Luopu Subdistrict | 2 | [Dongsha Subdistrict](https://en.wikipedia.org/w/index.php?title=Dongsha_Subdistrict&action=edit&redlink=1) | 1 |
| Yongping Subdistrict | 2 | [Chongkou Subdistrict](https://en.wikipedia.org/w/index.php?title=Chongkou_Subdistrict&action=edit&redlink=1) | 1 |
| **Beijing (11 June**–**5 July 2020)** | | | |
| Huaxiang Area | 192 | Changxindian town | 1 |
| [Xihongmen](https://en.wikipedia.org/w/index.php?title=Xihongmen,_Beijing&action=edit&redlink=1) Area | 25 | Changxindian Subdistrict | 1 |
| Xincun Subdistrict | 21 | [Yuetan Subdistrict](https://en.wikipedia.org/w/index.php?title=Yuetan_Subdistrict&action=edit&redlink=1) | 1 |
| [Huangcun](https://en.wikipedia.org/w/index.php?title=Huangcun,_Beijing&action=edit&redlink=1) Area | 17 | Youanmen Subdistrict | 1 |
| Yongdinglu Subdistrict | 10 | Yongdingmenwai Subdistrict | 1 |
| [Qingyuan Subdistrict](https://en.wikipedia.org/w/index.php?title=Qingyuan_Subdistrict,_Beijing&action=edit&redlink=1) | 9 | Yizhuang Area | 1 |
| Lugouqiao Area | 8 | [Xingfeng Subdistrict](https://en.wikipedia.org/w/index.php?title=Xingfeng_Subdistrict&action=edit&redlink=1) | 1 |
| Majiabao Subdistrict | 7 | Xiaohongmen Area | 1 |
| Tiancunlu Subdistrict | 6 | Wanshoulu Subdistrict | 1 |
| Nanyuan Subdistrict | 6 | [Tiantan Subdistrict](https://en.wikipedia.org/w/index.php?title=Tiantan_Subdistrict&action=edit&redlink=1) | 1 |
| Changyang town | 4 | Taipingqiao [Subdistrict](https://en.wikipedia.org/wiki/Hepingli_Subdistrict,_Beijing) | 1 |
| [Qingyundian](https://en.wikipedia.org/w/index.php?title=Qingyundian&action=edit&redlink=1) town | 4 | Sijiqing Area | 1 |
| Xiluoyuan Subdistrict | 3 | Shibalidian Area | 1 |
| [Weishanzhuang](https://en.wikipedia.org/w/index.php?title=Weishanzhuang&action=edit&redlink=1) town | 3 | Qinglongqiao Subdistrict | 1 |
| Nanyuan Area | 3 | [Panggezhuang](https://en.wikipedia.org/w/index.php?title=Panggezhuang&action=edit&redlink=1) town | 1 |
| Lugouqiao Subdistrict | 3 | [Lixian](https://en.wikipedia.org/w/index.php?title=Lixian,_Beijing&action=edit&redlink=1) town | 1 |
| Dahongmen Subdistrict | 3 | Jiugong Area | 1 |
| Zhanlan Road Subdistrict | 2 | Jinrong Street Subdistrict | 1 |
| Yongding Area | 2 | Huilongguan Area | 1 |
| Tiangongyuan Subdistrict | 2 | [Hepingli Subdistrict](https://en.wikipedia.org/wiki/Hepingli_Subdistrict,_Beijing) | 1 |
| Linxiao Road Subdistrict | 2 | Guang'anmenwai Subdistrict | 1 |
| Guanyinsi Subdistrict | 2 | Guang'anmennei Subdistrict | 1 |
| Fengtai Subdistrict | 2 | [Beizangcun](https://en.wikipedia.org/w/index.php?title=Beizangcun&action=edit&redlink=1) town | 1 |
| Beiyuan Subdistrict | 2 | Balizhuang [Subdistrict](https://en.wikipedia.org/w/index.php?title=Babaoshan_Subdistrict&action=edit&redlink=1) | 1 |
| Beixinqiao Subdistrict | 2 | [Babaoshan](https://en.wikipedia.org/w/index.php?title=Babaoshan_Subdistrict&action=edit&redlink=1) Subdistrict | 1 |
| [Baizhifang Subdistrict](https://en.wikipedia.org/w/index.php?title=Baizhifang_Subdistrict&action=edit&redlink=1) | 2 | [Anding](https://en.wikipedia.org/w/index.php?title=Anding,_Beijing&action=edit&redlink=1) town | 1 |

**Table S2. Average performance of mobility-based sampling under various outbreak and data scenarios in Guangzhou and Beijing.** Simulated data of different outbreak scenarios were generated by the constructed epidemiological model in both cities. Five types of scenarios were considered for each city, involving three basic reproduction numbers (R_0_), outbreaks beginning in high or low population-density communities, and two intervention timings. For each type, the size of the outbreak source was set to one/two/three communities. For example, the first row denotes randomly selecting one source community in Guangzhou where the communities with higher population density are more likely to be selected, with an R_0_ of 4.9 and the actual timing of non-pharmacological interventions. For each outbreak scenario, 30 simulations were repeated to test 30 random locations, and the table provides details on the proportion of estimated communities with infections (affected communities) and the performances of two mobility-based sampling approaches in detecting affected communities and cases for the mean of many random simulations.

| City | Scenario | | | |  | Simulated transmission | |  | | Average performance of sampling approach (%) | | | | |
| --- | --- | --- | --- | --- | --- | --- | --- | --- | --- | --- | --- | --- | --- | --- |
|  | Population density of source communities | Intervention timing | R_0_ | Number of source communities |  | Average proportion of affected communities (%) |  | | Case detection | | | Affected community detection | |  |
|  |  |  |  |  |  |  |  | | CFI | | CTI | CFI | CTI |  |
| Guangzhou | High | Actual | 4.9 | 1 |  | 21.1 |  | | 79.8 | | 66.2 | 84.3 | 67.6 |  |
|  |  |  |  | 2 |  | 51 |  | | 68.5 | | 57.5 | 71.6 | 59.4 |  |
|  |  |  |  | 3 |  | 68.6 |  | | 60.2 | | 48.4 | 63.2 | 52.6 |  |
|  | Low | Actual | 4.9 | 1 |  | 14.3 |  | | 88.8 | | 79.1 | 89 | 83.7 |  |
|  |  |  |  | 2 |  | 36.5 |  | | 78.4 | | 70.7 | 77.7 | 74 |  |
|  |  |  |  | 3 |  | 48.9 |  | | 72.8 | | 66.5 | 72 | 68.4 |  |
|  | High | Actual | 3.32 | 1 |  | 9.6 |  | | 83.3 | | 67.1 | 88.4 | 67.9 |  |
|  |  |  |  | 2 |  | 28.5 |  | | 73 | | 58.8 | 79.8 | 63.9 |  |
|  |  |  |  | 3 |  | 55.8 |  | | 62 | | 50.7 | 68.2 | 55.8 |  |
|  | High | Actual | 9.5 | 1 |  | 76 |  | | 60.4 | | 45.7 | 58.3 | 48.3 |  |
|  |  |  |  | 2 |  | 94.2 |  | | 55.4 | | 46.5 | 52.8 | 49.6 |  |
|  |  |  |  | 3 |  | 94.6 |  | | 55.1 | | 46.3 | 52.6 | 49.8 |  |
|  | High | One week delay | 4.9 | 1 |  | 65 |  | | 52.5 | | 46.6 | 61.5 | 58.1 |  |
|  |  |  |  | 2 |  | 87.7 |  | | 55.9 | | 47.3 | 55.2 | 52.9 |  |
|  |  |  |  | 3 |  | 92.7 |  | | 51.4 | | 45.5 | 53.5 | 49.9 |  |
| Beijing | High | Actual timing | 3.32 | 1 |  | 3 |  | | 98.3 | | 98.2 | 94.5 | 93.5 |  |
|  |  |  |  | 2 |  | 7 |  | | 97.5 | | 97.4 | 91.3 | 90.8 |  |
|  |  |  |  | 3 |  | 15.6 |  | | 94.6 | | 94.3 | 86.7 | 86.4 |  |
|  | Low | Actual timing | 3.32 | 1 |  | 2.2 |  | | 99.7 | | 99.6 | 98 | 97.4 |  |
|  |  |  |  | 2 |  | 4.1 |  | | 99.8 | | 99.6 | 96.5 | 95.8 |  |
|  |  |  |  | 3 |  | 6.5 |  | | 99.6 | | 99.4 | 94.8 | 93.9 |  |
|  | High | Actual timing | 4.9 | 1 |  | 5.9 |  | | 97.4 | | 97.3 | 91.3 | 90.2 |  |
|  |  |  |  | 2 |  | 12.6 |  | | 96.8 | | 96.5 | 88.4 | 88 |  |
|  |  |  |  | 3 |  | 18.9 |  | | 95.7 | | 95.4 | 85.9 | 85.7 |  |
|  | High | Actual timing | 9.5 | 1 |  | 31.2 |  | | 95.1 | | 94.8 | 81.7 | 80.7 |  |
|  |  |  |  | 2 |  | 46.3 |  | | 93.7 | | 93.2 | 75.4 | 75.2 |  |
|  |  |  |  | 3 |  | 55.7 |  | | 92 | | 91.1 | 71.5 | 71.4 |  |
|  | High | One week delay | 3.32 | 1 |  | 15.3 |  | | 93.8 | | 93.5 | 85.6 | 83.9 |  |
|  |  |  |  | 2 |  | 24.2 |  | | 93.8 | | 93.4 | 83 | 81.5 |  |
|  |  |  |  | 3 |  | 32.9 |  | | 92.3 | | 92 | 79.2 | 78.9 |  |

Abbreviations: R_0_ - basic reproduction number; CFI - case flow intensity; CTI - case transmission intensity.

**Table S3. Simulated transmission in Guangzhou and Beijing under various outbreak scenarios using spatial sampling approaches.** Spatial multiple rounds of mass testing were realized using spatial sampling and incorporated into the travel network-based epidemiological model (Supplementary Note 4). The disease transmission under various sampling approaches and outbreak scenarios, was simulated using the epidemiological model. The baseline approach of multi-round mass testing represented that daily testing resources were equally allocated to all communities within a city. However, SRS/CFI/CTI sampled a given number of communities per day and allocated more resources to sampled communities compared with those not sampled. Outbreaks under different settings were tested: basic reproduction numbers (R_0_) of original SARS-CoV-2, Delta, and Omicron variants, and the timing of interventions. On average, the proportion of affected communities over the total communities of a city and the ratio of cumulative cases using the spatial sampling to the cases using the baseline approach, were used to depict the simulated transmission.

| City | Scenario | |  | Simulated transmission | | | | | | | | |
| --- | --- | --- | --- | --- | --- | --- | --- | --- | --- | --- | --- | --- |
|  | R_0_ | Intervention timing |  | Average proportion of affected communities (%) | | | |  | Ratio of cases using the spatial sampling to cases using the baseline (%) | | | |
|  |  |  |  | Baseline | SRS | CFI | CTI |  | Baseline | SRS | CFI | CTI |
| Guangzhou | 4.9 | Actual |  | 37.3 | 32.4 | 26 | 31.8 |  | 100 | 90.6 | 80.7 | 86.1 |
|  | 9.5 |  |  | 71.3 | 65.4 | 62.8 | 67.1 |  | 100 | 87.1 | 72.2 | 83.2 |
|  | 4.9 | One-week delay |  | 68.1 | 64.8 | 63.2 | 65.8 |  | 100 | 92.6 | 87.7 | 89.9 |
|  | 9.5 |  |  | 94.3 | 93.2 | 93.7 | 92.5 |  | 100 | 89.9 | 80 | 83.8 |
| Beijing | 3.32 | Actual |  | 5.4 | 4.6 | 4.3 | 4.3 |  | 100 | 88.1 | 81.3 | 80.7 |
|  | 9.5 |  |  | 28 | 22.9 | 18.9 | 20.2 |  | 100 | 77.4 | 56.2 | 58.1 |
|  | 3.32 | One-week delay |  | 5.1 | 4.5 | 3.8 | 3.8 |  | 100 | 89.2 | 82.6 | 81.4 |
|  | 9.5 |  |  | 54.8 | 48.5 | 39.8 | 41.6 |  | 100 | 77.1 | 50.7 | 51.5 |

Abbreviations: R_0_ - basic reproduction number; SRS - simple random sampling; CFI - case flow intensity; CTI - case transmission intensity.

# Supplementary Texts

## Text S1: Mobile phone signaling and POI data

At the tower level, CSD was recorded when users made phone calls, sent messages, turned devices on/off, switched towers, etc. The data was aggregated to capture the movement of people between communities based on all users' mobile phone activity records throughout Beijing from 11 to 12 June 2020 and Guangzhou from 21 to 22 May 2021, respectively, during the early stage of COVID-19 outbreaks and before the implementation of travel restrictions across cities. Specifically, the data at the community level contains hourly origin-destination flow metrics. Users who stayed in a community for less than 15 minutes were filtered out to exclude people who only briefly passed by the community. The operator recorded the movement such that at a given hour, if a user was observed at communities A→B→C for more than 15 minutes, respectively, then A→B and B→C were counted. All mobile phone activity records were processed anonymously and aggregately, and it is impossible to identify or filter certain groups’ flows. Thus, the population flow presented in this study provides hourly and inter-regional flows of the general population and cannot be analyzed for individuals’ tracking.

The data in Beijing also contained hourly counts of the number of people who have been in a community (staying population). The user was counted if a user-generated CSD at the towers in the same community for 60 minutes. The comparison of the average hourly staying population during the night from 10 p.m. to 6 a.m. with the population extracted from WorldPop at communities in Beijing (Fig. S8), showed the high consistency between the staying population and WorldPop-extracted population. For example, the staying population accounted for nearly 68% (equal to the proportion of mobile customers) of the WorldPop-extracted population. The staying population had a 0.82 Pearson correlation with the WorldPop-extracted population, and the difference between the two types of populations was less than 50,000 persons in 81% of the communities.

A Point of Interest (POI) is a specific point location, or useful site, defined mainly by its geographical coordinates (longitude and latitude). It refers to a place or destination of potential interest and can be a tourist attraction, a hotel, a restaurant, an ATM, a pharmacy, a medical center, a store, a gas station, or any other category used in modern car navigation systems (https://www.korem.com/dictionary/point-of-interest-poi/). POI data obtained from the AMap Services used for the study contained six main core fields: POI name, multilevel categories, address, coordinate location, and district name. The AMap divided the POI into 23 primary categories, 241 secondary categories, and 2,035 tertiary categories. In terms of the number of all primary categories of POIs in Beijing and Guangzhou, locations for shopping were the most (Fig. S9a). Beyond that, POIs for daily life service, food & beverages, workplace, and place name/address were dominant. The diversity index for a community based on the POI number and category was calculated by extracting POIs located in the community (Fig. S9b). The communities with rich POI diversity in Guangzhou were scattered in various districts, while the POI diversity of the communities in the urban area of Beijing was higher than that of other communities.

**
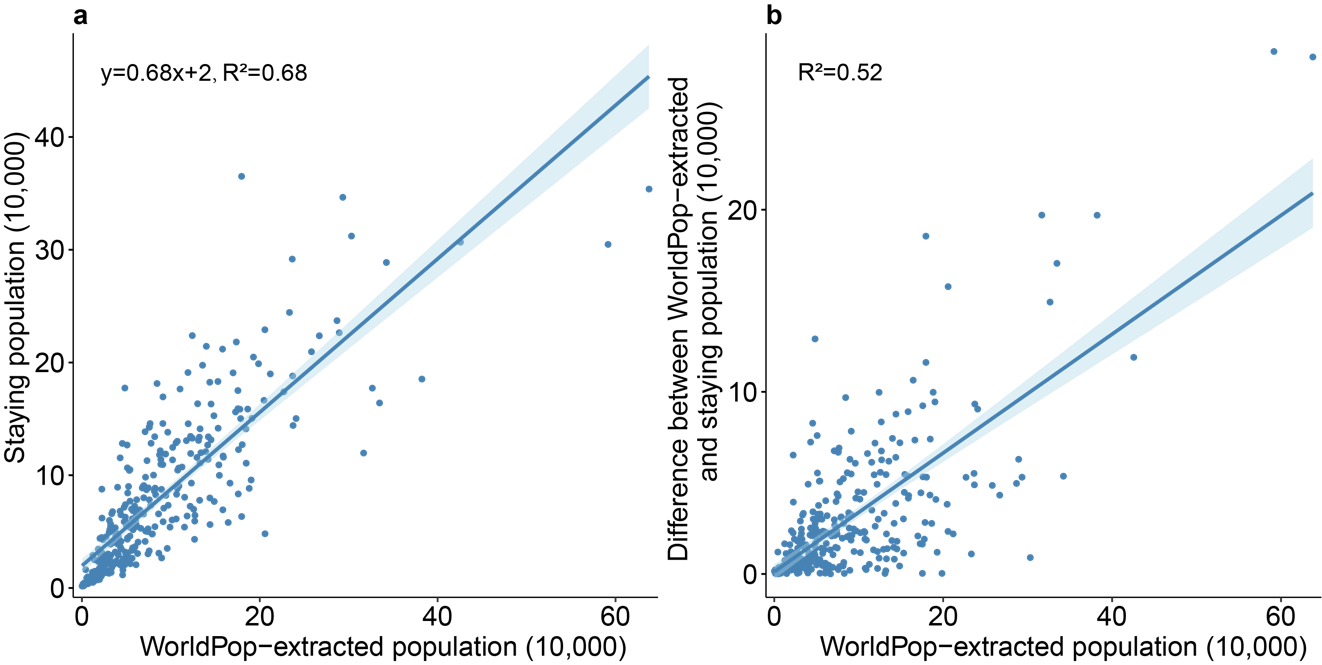
**

**Fig. S8. Comparison between the average hourly staying population from mobile phone signaling data and the population extracted from WorldPop in Beijing.** Staying populations (people who stayed in a community for 60 minutes) of the night time from 10 p.m. to 6 a.m. was hourly averaged. **a**, Linear regression between staying population and WorldPop-extracted population of communities in Beijing. **b**, Linear regression between the absolute value of the difference between these two types of populations and WorldPop-extracted population. Shaded regions represent 95% confidence intervals for the regressions.


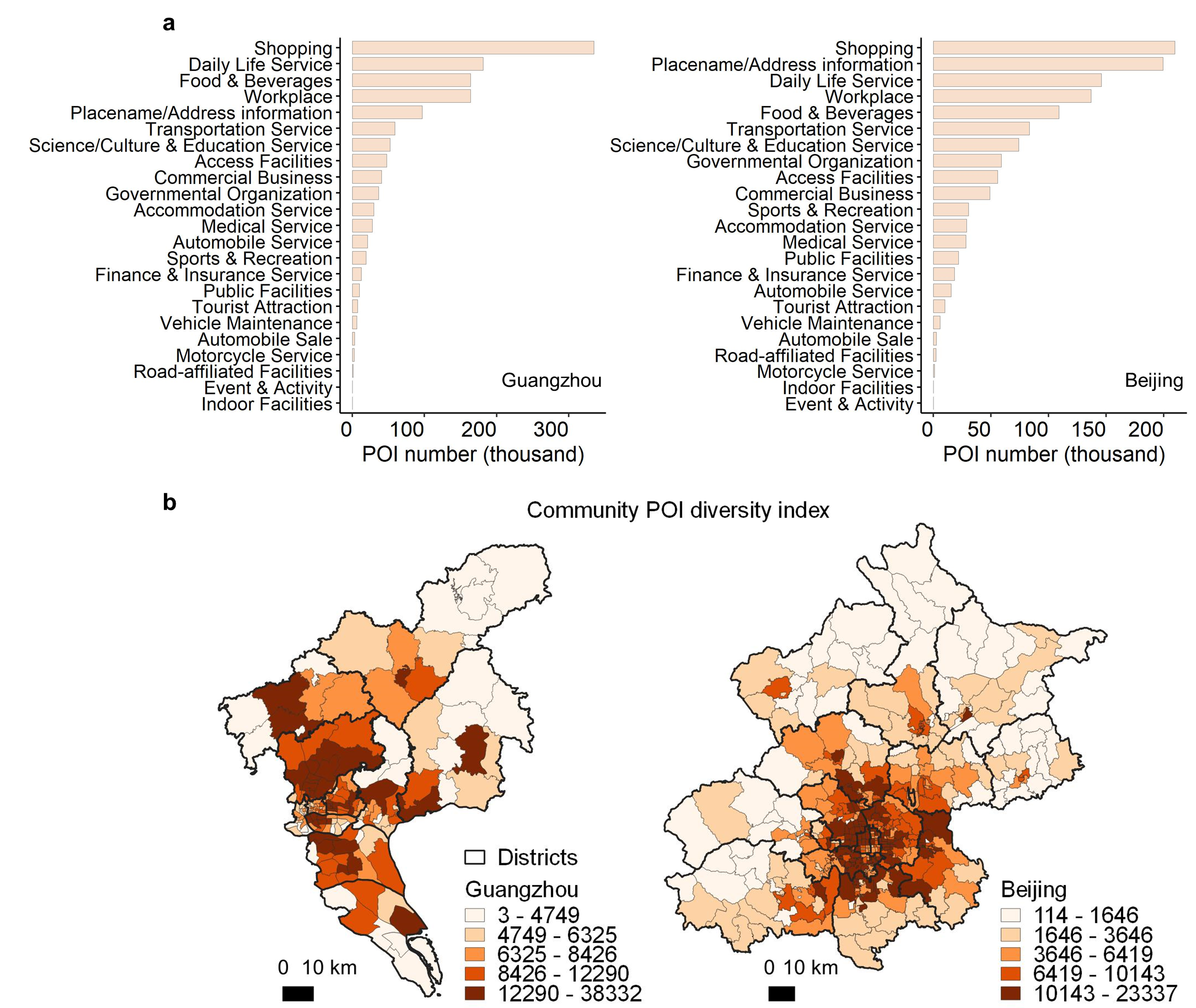


**Fig. S9. Number of POIs by category and POI diversity index for communities in Beijing and Guangzhou.** **a**, The number of POIs for 23 primary categories. **b**, The diversity index for a community based on the POI number and category was calculated by extracting POIs located in the community.

## Text S2: Constructing the SEIR model

A travel-network-based SEIR modeling framework (Lai et al. 2020) (<https://github.com/wpgp/BEARmod>), using human mobility data, was employed to estimate the COVID-19 spread in Guangzhou and Beijing, where the main parameters were determined in our study.

Epidemiological characteristics of SARS-CoV-2 Delta variant infections in Guangdong, China, from May to June 2021, have been explored in another study (Kang et al. 2022a). The mean incubation period was estimated at 5.8 days (95% CI: 5.1–6.5). Owing to 99.8% (93.2–100.0) of transmissions occurring within four days after illness onset, we calculated the daily contact rate using the basic reproduction number (R_0_=4.9, 3.1–6.5) divided by 4, weighted by the relative level of daily contact based on Baidu movement data (Baidu-based weight, Table S4). Patients infected with the Delta variant maintained a high viral load from four days before illness onset, and the number of days from illness onset to isolation of the first case in Guangzhou was two. We then use the initial number of days from infectiousness onset to isolation equal to 6. Considering the 5-category vaccination status (i.e., unvaccinated, intermediate 1^st^ dose, partially vaccinated, intermediate 2^nd^ dose, fully vaccinated), the average effectiveness of vaccine against infection for the outbreak in Guangzhou was equal to 13.9% (-4.7–32.5) (Kang et al. 2022b). The start date of the SEIR model simulation was set to 13 May 2021, considering the first case with symptoms on 18 May 2021 and the mean incubation period of 5.8 days.

In terms of the epidemiological parameters for the COVID-19 outbreak in Beijing's Xinfadi Market (Luo et al. 2022), the incubation period was assumed to have a mean of 5.2 days (4.1–7.0) (Li et al. 2020). Due to the high transmissibility during the first five days after illness onset (Byrne et al. 2020), we calculated the daily contact rate using the basic reproduction number (R_0_=3.32, 1.4–3.9 (Byambasuren et al. 2020)) divided by 5, weighted by the daily Baidu-based weight (Table S4). Infectiousness was apparent in an average of two to three days prior to the development of symptoms (He et al. 2020), and the duration from illness onset to isolation of the first case in Beijing was five days. Therefore, the initial lags from infectiousness onset to isolation were set to 7.5 days. The start date of simulation was set to 3 June 2020, due to that the first case went to the Xinfadi Market on that day.

For the outbreaks in Guangzhou and Beijing, we used time lags from the first day of infectiousness period to the date of isolation as the proxy of the infectious period. It was shortened with the implementation of large-scale nucleic acid testing that was used to infer the changing timeliness of case isolation across the outbreak (Table S5). A total of 73.3% of people demonstrated good compliance with face mask use during the COVID-19 pandemic (Tan et al. 2021), and the proportion of daily mask use changed during the transmission according to the daily Baidu search index, using “mask” as a search term (<https://index.baidu.com/>). Moreover, the probability of being infected for people with face masks was 1/1.44 (1/1.82–1/1.24) compared with that for people without masks (Andrejko et al. 2022). The efficacy of wearing masks in reducing transmission could be computed according to the probability and the percentage of the wearing-mask populations (Howard et al. 2021).

The mean of many simulations (e.g., 500) using the SEIR model for the cumulative number of cases was regarded as the community-level infection risk, and affected communities were estimated during an outbreak by rounding up the infection risk. The indicators, sensitivity and specificity, were defined for depicting the spatial accuracy of the estimates by the constructed SEIR for the outbreaks in Guangzhou and Beijing (Table S6).

**Table S4.** Daily Baidu-based weight during COVID-19 outbreaks in Guangzhou and Beijing. Baidu-based weight was computed using the counts of travelers during the outbreak divided by the average level before the pandemic (1-21 January 2020), based on Baidu movement data.

| **Guangzhou** | |  | **Beijing** | |
| --- | --- | --- | --- | --- |
| Date | Baidu-based weight |  | Date | Baidu-based weight |
| 2021/5/21 | 0.51 |  | 2020/6/11 | 0.50 |
| 2021/5/22 | 0.68 |  | 2020/6/12 | 0.61 |
| 2021/5/23 | 0.49 |  | 2020/6/13 | 0.37 |
| 2021/5/24 | 0.52 |  | 2020/6/14 | 0.39 |
| 2021/5/25 | 0.43 |  | 2020/6/15 | 0.34 |
| 2021/5/26 | 0.42 |  | 2020/6/16 | 0.33 |
| 2021/5/27 | 0.40 |  | 2020/6/17 | 0.37 |
| 2021/5/28 | 0.44 |  | 2020/6/18 | 0.51 |
| 2021/5/29 | 0.53 |  | 2020/6/19 | 0.35 |
| 2021/5/30 | 0.38 |  | 2020/6/20 | 0.25 |
| 2021/5/31 | 0.40 |  | 2020/6/21 | 0.26 |
| 2021/6/1 | 0.31 |  | 2020/6/22 | 0.19 |
| 2021/6/2 | 0.28 |  | 2020/6/23 | 0.24 |
| 2021/6/3 | 0.26 |  | 2020/6/24 | 0.25 |
| 2021/6/4 | 0.28 |  | 2020/6/25 | 0.31 |
| 2021/6/5 | 0.30 |  | 2020/6/26 | 0.32 |
| 2021/6/6 | 0.24 |  | 2020/6/27 | 0.20 |
| 2021/6/7 | 0.26 |  | 2020/6/28 | 0.23 |
| 2021/6/8 | 0.22 |  | 2020/6/29 | 0.24 |
| 2021/6/9 | 0.22 |  | 2020/6/30 | 0.26 |
| 2021/6/10 | 0.22 |  | 2020/7/1 | 0.25 |
| 2021/6/11 | 0.22 |  | 2020/7/2 | 0.29 |
| 2021/6/12 | 0.32 |  | 2020/7/3 | 0.34 |
| 2021/6/13 | 0.27 |  | 2020/7/4 | 0.23 |
| 2021/6/14 | 0.23 |  | 2020/7/5 | 0.25 |
| 2021/6/15 | 0.25 |  |  |  |
| 2021/6/16 | 0.23 |  |  |  |
| 2021/6/17 | 0.22 |  |  |  |
| 2021/6/18 | 0.24 |  |  |  |

**Table S5.** Daily infectious period for COVID-19 outbreaks in Guangzhou and Beijing. The infectious period represents time lags from infectiousness onset to isolation. The implementation of large-scale nucleic acid testing was used to infer the changing timeliness of case isolation across the outbreak, and the minimum of the infectious period was two days.

| **Guangzhou** | |  | **Beijing** | |
| --- | --- | --- | --- | --- |
| Date | Infectious period (days) |  | Date | Infectious period (days) |
| 2021/5/20 | 6 |  | 2020/6/10 | 7.5 |
| 2021/5/21 | 5 |  | 2020/6/11 | 6 |
| 2021/5/22 | 5 |  | 2020/6/12 | 5 |
| 2021/5/23 | 5 |  | 2020/6/13 | 5 |
| 2021/5/24 | 5 |  | 2020/6/14 | 5 |
| 2021/5/25 | 5 |  | 2020/6/15 | 5 |
| 2021/5/26 | 4 |  | 2020/6/16 | 5 |
| 2021/5/27 | 4 |  | 2020/6/17 | 4 |
| 2021/5/28 | 4 |  | 2020/6/18 | 4 |
| 2021/5/29 | 4 |  | 2020/6/19 | 4 |
| 2021/5/30 | 3 |  | 2020/6/20 | 4 |
| 2021/5/31 | 3 |  | 2020/6/21 | 4 |
| 2021/6/1 | 3 |  | 2020/6/22 | 4 |
| 2021/6/2 | 3 |  | 2020/6/23 | 3 |
| 2021/6/3 | 3 |  | 2020/6/24 | 3 |
| 2021/6/4 | 2 |  | 2020/6/25 | 3 |
| 2021/6/5 | 2 |  | 2020/6/26 | 2 |

**Table S6.** The confusion matrix for the classification of communities with and without cases between estimated and reported transmissions for COVID-19 outbreaks in Guangzhou and Beijing. The ratio of accurate estimates of affected communities over actual communities with cases was expressed as sensitivity, and the ratio of accurate estimates of communities without cases over actual communities with no cases was also defined as specificity. The number of estimated affected communities was presented as the mean of 500 simulations.

| City | Estimated  Reported | Affected communities | Communities without COVID-19 | Total |
| --- | --- | --- | --- | --- |
| Guangzhou | Affected communities | 13 | 3 | 16 |
|  | Communities without COVID-19 | 12 | 140 | 152 |
|  | Total | 25 | 143 | 168 |
|  |  | Sensitivity: 0.81; Specificity:0.92 | | |
| Beijing | Affected communities | 28 | 24 | 52 |
|  | Communities without COVID-19 | 13 | 266 | 279 |
|  | Total | 41 | 290 | 331 |
|  |  | Sensitivity: 0.54; Specificity:0.95 | | |

## Text S3: Uncertainty of infection risk assessment

Community-level infection risks in each mobility-based sampling approach were estimated under various runs with different settings. A total of 50 runs were conducted for the HCI, where the exponential factor ($q$) of the POI diversity index was set to the values of [0, 0.02, ..., 0.98, 1]. With 48 runs tested for the HFI, the duration ($T$) was set to 48 values of [1, 2, …, 47, 48] to generate cumulative volume of hourly population flow based on the two-day mobility data before the stringent non-pharmaceutical interventions were implemented. In addition, because people might be infected or spread the disease before the implementation of strict travel restrictions and mass testing, multiple runs for CFI and CTI were tested using different selections of initial cases over time in Guangzhou and Beijing, respectively (Table S7). The SEIR also estimated community-level infection risk by the mean of 500 simulations, and we repeated the process 100 times to obtain SEIR-informed infection risks under 100 runs. For each sampling approach, the mean and 95% confidence interval were computed in terms of infection risks under many runs.

**Table S7.** Selection of initial cases from reported cases in different periods for the mobility-based case flow intensity (CFI) and case transmission intensity (CTI) sampling over time in Guangzhou and Beijing.

| **City** | **Mobility data** | **Period of cases selected** |
| --- | --- | --- |
| Guangzhou | 21–22 May 2021 | 21–25 May 2021 |
|  |  | 21–26 May 2021 |
|  |  | 21–27 May 2021 |
|  |  | 25 May 2021 |
|  |  | 25–26 May 2021 |
|  |  | 25–27 May 2021 |
|  |  | 26–27 May 2021 |
| Beijing | 11–12 June 2020 | 11–13 June 2020 |
|  |  | 11–14 June 2020 |
|  |  | 11–15 June 2020 |
|  |  | 12–13 June 2020 |
|  |  | 12–14 June 2020 |
|  |  | 12–15 June 2020 |
|  |  | 13–14 June 2020 |
|  |  | 13–15 June 2020 |

## Text S4: Two ways of determining sampled communities

Taking the community in a city as the sampling unit, optimized spatial sampling approaches were achieved in two ways. Given sample size equal to $n$, the first one was the deterministic way: community-level infection risk ($\rho$) was ranked from high to low, and the top $n$ communities in terms of the highest $\rho$ were selected as the sample. The second was a Poisson way (Williams et al. 2009): a random number ($\in Uniform(0,1)$) was generated for each community, and the community was sampled with the random number less than the community’s probability which was given by $\pi_{i}=\frac{n\cdot\rho_{i}}{\sum_{i=1}^{N} \rho_{i}}$, where $\sum_{i=1}^{N} \rho_{i}$ was the sum of $\rho$ of all communities in a city. Under given sample size, sampled communities were the same in the deterministic way, while the sample in the Poisson way might differ each time due to the randomness.

## Text S5: Sensitivity analyses for mobility scenarios and SEIR parameter combinations

Performances of mobility-based spatial sampling were tested in simulated outbreaks under various mobility contributions. Figs. 2d and 2h showed the real-world inter-community mobility pattern in Guangzhou and Beijing, respectively. There were movements among most communities in Guangzhou, even between two communities far away across different districts, while inter-community movements in Beijing were mainly intra-district movements. On average, the people in one community went to 96.6% of Guangzhou’s communities in one day. The number of communities to which people went from one community accounts for 59.4% in Beijing (Fig. S3a). Under the same initial settings, simulated outbreaks affected a larger proportion of Guangzhou communities than in Beijing. The average performance of CFI and CTI was higher in Beijing for improving the efficiency of detecting infections (Fig. S2). We exchanged the inter-community mobility patterns of Beijing and Guangzhou to assess CFI and CTI under different spatial patterns of human movements. For instance, the human mobility pattern in Guangzhou was generated in terms of Beijing’s real-world mobility characteristics (Figs. S3b-3c). Using the constructed SEIR model based on simulated inter-community mobility data in a city, we generated epidemics under two R_0_ to assess CFI and CTI (Figs. S3d-3e). We found that the performances of CFI and CTI were superior for the outbreaks in Guangzhou, even for high-transmission scenarios (R_0_ = 9.5). In addition, CFI and CTI improved the efficiency of case identification for simulated outbreaks in Beijing, while their performances decreased for the scenarios with higher R_0_.

We also assessed CFI and CTI in simulated outbreaks under various levels of population mobility in Guangzhou and Beijing (Fig. S4). The levels of population mobility changed for the top 20% of real-world inter-community flows within a city, and the change rates were 0%, 50%, 150%, and 200%, respectively. Based on the mobility data, by increasing/decreasing the volume of high-impact inter-community flows, SEIR simulated outbreaks with R_0_ equal to 4.9 in Guangzhou and 3.32 in Beijing. Overall, CFI and CTI outperformed SRS under different levels of flow volume, while their effectiveness decreased with increased mobility. Nevertheless, the reduction of the effectiveness of CFI and CTI was less when the mobility increased to a certain extent; for example, there was little difference for the flows increasing to 150% and 200%.

Mobility-based spatial sampling is an unsupervised method, and the estimated results mainly depend on the mobility data and the case data in the initial stage. The data from two cities were used in our study, but the proposed method can directly reveal the possible epidemic situation through the travel pattern in the early stage. A series of sensitivity analyses illustrated that the efficiency of detecting emerging infections could be improved effectively using the proposed method in the early stage for actual and simulated outbreaks under various mobility patterns.

CFI and CTI have been assessed by many simulated outbreaks, considering various initial locations of disease emergence, transmissibility, population density, and mobility-mediated spread within a city over time (Fig. 4). However, the SEIR model estimated the outbreaks using the same daily parameters. We further assessed CFI and CTI using SEIR with random combinations of R_0_, incubation period, and infectious period. Specifically, multiple candidates were produced for each parameter in terms of its maximum and minimum described in Text S1, that is, R_0_ changing daily from 1.4 to 6.5 (at 0.1 intervals), incubation period between 4.1 and 7, and infectious period based on mean and variance. The SEIR model randomly selected one from the range per day for each parameter. For simulated outbreaks under various initial source locations using SEIR with different parameter combinations, CFI and CTI could improve the efficiency of capturing infections. Their performances were better for outbreaks in Beijing than Guangzhou (Fig. S5).

## Text S6: Incorporating spatial sampling into the SEIR for multi-round testing

We incorporated spatial sampling into the SEIR to simulate disease transmission under multiple rounds of mass testing, where a baseline, simple random sampling (SRS), CFI, and CTI approaches were considered.

First, the baseline approach of multi-round mass testing represented that daily testing resources were equally allocated to all communities within a city. It was achieved at the same contact rate for all communities, and the contact rate was consistent to that used for the constructed SEIR (Supplementary Note 1). Specifically, the contact rate on day $d$ for each community was computed using R_0_ divided by the high-transmissibility period after illness onset (4 for Guangzhou and 5 for Beijing), weighted by the Baidu-based weight on day $d$ (denoted by $b_{d}$). For the baseline, we expressed the contact rate on day $d$ as $C_{d}^{base}$, and used $C_{d}^{base}\cdot N$ to depict the level of daily testing resources in total for $N$ communities.

However, SRS, CFI, and CTI sampled a given number of communities per day, and allocated more resources to sampled communities than unsampled areas. Each community had the same probability of be sampled using the SRS, while a higher infection-risk community would have a greater probability of being sampled using the CFI or CTI. Supposing that $n$ communities were sampled on day $d$, the contact rate for sampled communities would be reduced compared to the baseline. The decline rate was equal to the reciprocal of time lags from exposure to contagiousness ($l_{i2i}$), and the contact rate on day $d$ was given by $C_{d}^{base}\cdot\frac{1}{l_{i2i}}$. The contact rate for unsampled communities was defined as $C_{d}^{base}\cdot(1+\left( 1-\frac{1}{l_{i2i}} \right)\cdot\frac{n}{N-n})$. The sum of the contact rate for all communities was also $C_{d}^{base}\cdot N$, which guaranteed equivalent total testing resources for different approaches.

Citywide screening for the real-world outbreak in Guangzhou was launched on the fifth day of the outbreak and covered all communities after 12 days. For the simulated disease transmission in Guangzhou and Beijing, we assumed that mass testing was conducted on the fifth day of an outbreak and last for 12 days. Moreover, 1/12 of all communities was sampled each day, which approximates the maximum of test capacity for Guangzhou (i.e., 8% (CGTN 05-Jun-2021)). Specifically, we firstly used SEIR to generate the epidemiological data of the first four days of an outbreak, and community-level infection risks were obtained using CFI and CTI based on mobility data for the first two days of an outbreak and initial cases. Second, using CFI and CTI to sample the communities based on the infection risks on the fifth day, and the SEIR model estimates the new infections on the day at communities with different contact rate. Then, the process was repeated per day, and a community might be sampled many times in 12-days mass testing. According to parameters in Supplementary Note 1, the number of time lags from exposure to contagiousness was 1.8 (1.1–2.5) for Guangzhou and 2.7 (1.6–4.5) for Beijing.

# References

Andrejko, K.L., Pry, J.M., Myers, J.F., Fukui, N., & DeGuzman, J.L. (2022). Effectiveness of Face Mask or Respirator Use in Indoor Public Settings for Prevention of SARS-CoV-2 Infection — California, February–December 2021. In, *MMWR Morb Mortal Wkly Rep 2022* (pp. 212-216)

Byambasuren, O., Cardona, M., Bell, K., Clark, J., McLaws, M.-L., & Glasziou, P. (2020). Estimating the extent of asymptomatic COVID-19 and its potential for community transmission: Systematic review and meta-analysis. *Official Journal of the Association of Medical Microbiology and Infectious Disease Canada, 5*, 223-234

Byrne, A.W., McEvoy, D., Collins, A.B., Hunt, K., Casey, M., Barber, A., Butler, F., Griffin, J., Lane, E.A., McAloon, C., Brien, K., Wall, P., Walsh, K.A., & More, S.J. (2020). Inferred duration of infectious period of SARS-CoV-2: rapid scoping review and analysis of available evidence for asymptomatic and symptomatic COVID-19 cases. *BMJ Open, 10*, e039856

CGTN (05-Jun-2021). Guangzhou builds the most capable COVID-19 testing lab in 10 hours. In, *CGTN*. China: CGTN

He, X., Lau, E.H.Y., Wu, P., Deng, X., Wang, J., Hao, X., Lau, Y.C., Wong, J.Y., Guan, Y., Tan, X., Mo, X., Chen, Y., Liao, B., Chen, W., Hu, F., Zhang, Q., Zhong, M., Wu, Y., Zhao, L., Zhang, F., Cowling, B.J., Li, F., & Leung, G.M. (2020). Temporal dynamics in viral shedding and transmissibility of COVID-19. *Nature Medicine, 26*, 672-675

Howard, J., Huang, A., Li, Z., Tufekci, Z., Zdimal, V., van der Westhuizen, H.-M., von Delft, A., Price, A., Fridman, L., Tang, L.-H., Tang, V., Watson Gregory, L., Bax Christina, E., Shaikh, R., Questier, F., Hernandez, D., Chu Larry, F., Ramirez Christina, M., & Rimoin Anne, W. (2021). An evidence review of face masks against COVID-19. *Proceedings of the National Academy of Sciences, 118*, e2014564118

Kang, M., Xin, H., Yuan, J., Ali, S.T., Liang, Z., Zhang, J., Hu, T., Lau, E.H., Zhang, Y., Zhang, M., Cowling, B.J., Li, Y., & Wu, P. (2022a). Transmission dynamics and epidemiological characteristics of SARS-CoV-2 Delta variant infections in Guangdong, China, May to June 2021. *Eurosurveillance, 27*, 2100815

Kang, M., Yi, Y., Li, Y., Sun, L., Deng, A., Hu, T., Zhang, J., Liu, J., Cheng, M., Xie, S., Luo, M., Jiang, J., Jiang, Y., Tang, S., & He, J. (2022b). Effectiveness of Inactivated COVID-19 Vaccines Against Illness Caused by the B.1.617.2 (Delta) Variant During an Outbreak in Guangdong, China. *Annals of Internal Medicine, 175*, 533-540

Lai, S., Ruktanonchai, N.W., Zhou, L., Prosper, O., Luo, W., Floyd, J.R., Wesolowski, A., Santillana, M., Zhang, C., Du, X., Yu, H., & Tatem, A.J. (2020). Effect of non-pharmaceutical interventions to contain COVID-19 in China. *Nature, 585*, 410-413

Li, Q., Guan, X., Wu, P., Wang, X., Zhou, L., Tong, Y., Ren, R., Leung, K.S.M., Lau, E.H.Y., Wong, J.Y., Xing, X., Xiang, N., Wu, Y., Li, C., Chen, Q., Li, D., Liu, T., Zhao, J., Liu, M., Tu, W., Chen, C., Jin, L., Yang, R., Wang, Q., Zhou, S., Wang, R., Liu, H., Luo, Y., Liu, Y., Shao, G., Li, H., Tao, Z., Yang, Y., Deng, Z., Liu, B., Ma, Z., Zhang, Y., Shi, G., Lam, T.T.Y., Wu, J.T., Gao, G.F., Cowling, B.J., Yang, B., Leung, G.M., & Feng, Z. (2020). Early Transmission Dynamics in Wuhan, China, of Novel Coronavirus–Infected Pneumonia. *New England Journal of Medicine, 382*, 1199-1207

Luo, T., Wang, J., Wang, Q., Wang, X., Zhao, P., Zeng, D.D., Zhang, Q., & Cao, Z. (2022). Reconstruction of the Transmission Chain of COVID-19 Outbreak in Beijing's Xinfadi Market, China. *International Journal of Infectious Diseases, 116*, 411-417

Tan, M., Wang, Y., Luo, L., & Hu, J. (2021). How the public used face masks in China during the coronavirus disease pandemic: A survey study. *International Journal of Nursing Studies, 115*, 103853

Williams, M.S., Ebel, E.D., & Wells, S.J. (2009). Poisson sampling: A sampling strategy for concurrently establishing freedom from disease and estimating population characteristics. *Preventive Veterinary Medicine, 89*, 34-42
